# 2138. Evaluating the Safety and Immunogenicity of the rZIKV/D4Δ30-713 Live Attenuated Chimeric Zika Candidate Vaccine in Healthy Flavivirus-Naïve Adults

**DOI:** 10.1093/ofid/ofac492.1758

**Published:** 2022-12-15

**Authors:** Diana Zhong, Beth Kirkpatrick, Kristen Pierce, Urvi Patel, Madeleine R Blunt, Anna Durbin

**Affiliations:** Johns Hopkins University School of Medicine, Baltimore, Maryland; University of Vermont College of Medicine, Burlington, Vermont; University of Vermont College of Medicine, Burlington, Vermont; Johns Hopkins University Bloomberg School of Public Health, Baltimore, Maryland; Johns Hopkins University, Baltimore, Maryland; Johns Hopkins Bloomberg School of Public Health, Baltimore, Maryland

## Abstract

**Background:**

Zika virus (ZIKV) is a flavivirus associated with a serious congenital Zika syndrome, with a recent epidemic originating in Brazil in 2015. Novel ZIKV vaccine candidates are in development. We report results of a phase I study of the first live attenuated ZIKV vaccine for use in humans. rZIKV/D4Δ30-713 is a chimeric Zika vaccine, expressing the premembrane (prM) and envelope (E) genes of a contemporary ZIKV strain within a dengue DEN4Δ30 background.

**Methods:**

We conducted a phase I, randomized, placebo-controlled double-blind trial, evaluating two different doses, 10^3^ PFU and 10^4^ PFU, of rZIKV/D4Δ30 in healthy adult subjects 18 – 50. Between October 2018 and August 2021, 28 subjects were enrolled in each dose cohort (20 to vaccine; 8 to placebo) at the Johns Hopkins Center for Immunization Research and the University of Vermont Vaccine Testing Center. Subjects were followed 26 weeks post-vaccination. Primary outcomes included safety and reactogenicity (adverse events graded by severity) and immunogenicity assessed by seroconversion by day 90 post-vaccination (ZIKV peak titer ≥ 1:10). The study was IRB approved (WCG 20181303, UVM CHRMS 18-0566).

**Results:**

Fifty-five of 56 enrolled subjects completed the study. One volunteer (10^4^ PFU group) withdrew before study day 28 due to work conflicts. The most common adverse events were headache, fatigue, and myalgias. These events did not occur significantly more frequently in vaccine recipients compared with placebo recipients. One severe adverse event was deemed unrelated to study product (new breast cancer diagnosis). In the 10^3^ PFU group, 9 of 20 (45%) seroconverted. In the 10^4^ PFU group, 7 of 19 (37%) seroconverted (Figure 1). ZIKV could not be recovered by culture or PCR in the blood, saliva, or urine from any subject.

**Figure:**
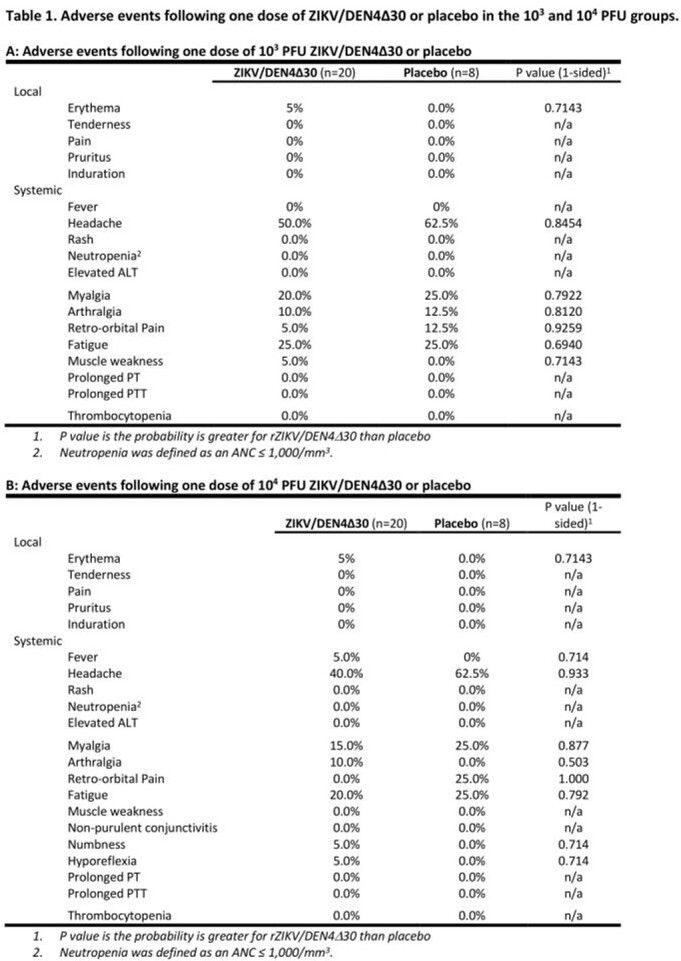

**Figure:**
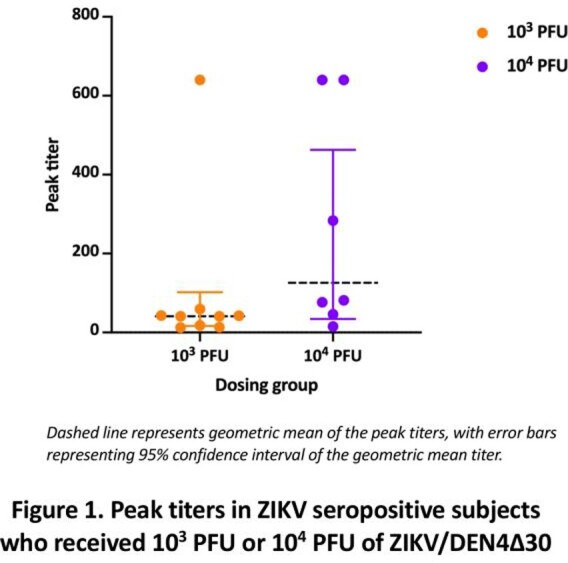

**Conclusion:**

The vaccine appears to be safe and well-tolerated in healthy flavivirus-naïve adults. The seroconversion proportion was similar between the two dosing groups, with higher peak titers in the 10^4^ PFU group. Given the lack of dose effect on seroconversion, further dose increases are unlikely to improve seroconversion. Chimerization can be highly attenuating to viruses. Our results suggest rZIKV/DEN4Δ30 is over-attenuated and thus will not be further developed as a candidate ZIKV vaccine.

**Disclosures:**

**Anna Durbin, MD**, Merck & Co.: Advisor/Consultant.

